# A 627K variant in the PB2 protein of H9 subtype influenza virus in wild birds

**DOI:** 10.1111/irv.12592

**Published:** 2018-09-12

**Authors:** Ye Ge, Qiucheng Yao, Hongliang Chai, Yuping Hua, Guohua Deng, Hualan Chen

**Affiliations:** ^1^ Guangdong Ocean University Zhanjiang Guangdong Province China; ^2^ State Key Laboratory of Veterinary Biotechnology Harbin Veterinary Research Institute of Chinese Academy of Agricultural Sciences Harbin China; ^3^ College of Wildlife Resources Northeast Forestry University Harbin Heilongjiang Province China

**Keywords:** epidemiology, H9N2 subtype, influenza virus, phylogeny

## Abstract

**Background:**

Wild birds are gaining increasing attention as gene‐mixing reservoirs for influenza viruses. To investigate the molecular properties of the viruses isolated and epidemiological analysis of H9N2 subtype AIV in wild birds, we studied samples obtained over two years (2014‐2015) from wetlands in Anhui province, China.

**Methods:**

A total of 4534 samples were collected from migratory waterfowl in Anhui in 2014‐2015, and 8 strains of H9 subtype AIV were isolated.

**Results:**

Phylogenetic analysis showed different degrees of gene segment reassortment in H9 viruses between the Eurasian lineage and the North American lineage. Most importantly, two viruses harbored the E627K mutation in the polymerase PB2 (PB2) protein. This is the first report of the mutation of this virus from low pathogenicity to high pathogenicity in wild birds.

**Conclusions:**

The continued surveillance of wild birds, especially migratory birds, is important to provide early warning and control of AIV outbreaks. Our results highlight the high genetic diversity of AIV along the Eurasian‐Australian migration flyway and the need for more extensive AIV surveillance in eastern China.

## 1 INTRODUCTION

Influenza A virus in wild birds includes the 16 HA and 9 NA types,^1^ and wild birds are gaining increasing attention as gene‐mixing reservoirs for influenza viruses.^2^ Wild bird origin avian influenza viruses (AIVs) may gain the ability to infect domestic poultry and humans more effectively after reassortment with one or more AIVs that are already well adapted to domestic birds or have certain amino acid alterations that are adaptive to mammals.^3^

Humans and animals are threatened by influenza A virus because of its frequent changes via mutation, recombination, and/or reassortment. H10N8 and H7N9, which may be fatal in human, reassortment among the hemagglutinin (HA), neuraminidase (NA), and internal genes of H9N2 have been observed, along with the E627K mutation in the polymerase PB2 protein.^4^, ^5^, ^6^, ^7^ H9N2 is widespread in nature and is sporadically detected in many poultry and even human beings.^8^, ^9^ After the isolation of the first strain in 1994, H9N2 AIVs have rapidly differentiated such that more than 102 genotypic variants have now been recognized based on the nomenclature system.^10^ In this study, we describe the detection and genetic characteristics of H9N2 viruses in wild birds in Anhui province in 2014‐2015. We successfully acquired eight H9N2 viruses, two of which harbored the E627K mutation in their PB2 protein. Our results provide baseline information about the prevalence of H9N2 AIVs in wild birds. This study found that influenza A virus wildly exist in wild birds and had the potential threat to mammal or human beings.

## 2 MATERIAL AND METHODS

### 2.1 Virus isolation and identification

In 2014‐2015, 4534 samples, including feces, oral swabs, and cloacal swabs, were collected from wild birds in Anhui province, China. The samples were placed in a phosphate‐buffered solution (pH 7.0), which included penicillin, streptomycin, and 10% glycerin, and were stored at a low temperature for transport. After the samples were prepared, they were inoculated into 9‐day‐old specific pathogen‐free (SPF) chicken embryos, and allantoic fluid was harvested after 72 hours of culture. The HA activity of the allantoic fluid harvested from each generation was evaluated using an HA test. Hemagglutination inhibition (HI) tests were also carried out with H1‐H16 monofactor serum. The HA activity and HI test results were verified by subtype‐specific real‐time PCR (RT‐PCR). NA subtypes were directly analyzed by subtype‐specific RT‐PCR and sequencing analysis.

Viral RNA was extracted from the allantoic fluid samples that were positive in the HA test using TRIzol (Invitrogen, Carlsbad, CA, USA). RNA was reverse‐transcribed into cDNA, which was amplified by PCR with primers complementary to the conserved promoter and noncoding region of each gene segment (Table 1). The PCR mix contained 1 μL of cDNA, 1 μL of forward primer and reverse primer, 5 μL of 10× *Taq* buffer (TaKaRa Bio Group, Dalian, Japan), 4 μL of 2.5 mmol/L dNTPs (TaKaRa Bio Group), 1 μL of Ex Taq (TaKaRa Bio Group), and 37 μL of RNase‐free water for a final volume of 50 μL. A single PCR program was used for all primers: initial denaturation at 95°C for 10 minutes; 30 cycles of 95°C for 30 seconds, 56°C for 30 seconds, and 72°C for 1.5 minutes; and a final extension at 72°C for 10 minutes. The PCR products were purified using a PCR purification kit (Tian Gen, Beijing, China) and sequenced on an Applied Biosystems DNA analyzer (ABI3500S; Applied Biosystems, Foster City, CA, USA).

**Table 1:** The viruses‘characters of H9N2 avian influenza virus

No.	Virus Name	Abbreviation	Time	Place	Host
1	A/Anseriformes**/**Anhui/S66/2014(H9N2)	AH/S66	2014.12	Anhui Province, Shengjin Lake	Anseriformes
2	A/Anseriformes**/**Anhui/S93/2014(H9N2)	AH/S93	2014.12	Anhui Province, Shengjin Lake	Anseriformes
3	A/Anseriformes/Anhui/S102/2014(H9N2)	AH/S102	2014.12	Anhui Province, Shengjin Lake	Anseriformes
4	A/Anseriformes/Anhui/L258/2014(H9N2)	AH/L258	2014.12	Anhui Province, Caizi Lake	Anseriformes
5	A/Anseriformes/Anhui/L281/2014(H9N2)	AH/L281	2014.12	Anhui Province, Caizi Lake	Anseriformes
6	Apigeon faecesAnhuiDGG4 2015(H9N2)	AH/DGG4	2015.03	Anhui Province, Digou district	Pigeon
7	AAnseriformes Anhui BLH12 2015(H9N2)	AH/BLH12	2015.03	Anhui Province, Bali river	Anseriformes

### 2.2 Genetic and phylogenetic analyses

Nucleotide sequences were edited using the SeqMan module of the DNAstar package, and phylogenetic analyses were performed with MAGE 6.0 maximum likelihood trees. Multiple sequence alignments were compiled using Clustal W. Phylogenetic analyses were based on the following coding sequences (nucleotides): PB2, 1‐2280; polymerase PB1 (PB1), 1‐2274; polymerase PBA (PA), 1‐2151; HA, 1‐1701; nucleoprotein (NP), 1‐1497; NA, 1‐1410; matrix protein 1 (M), 1‐982; and nonstructural protein 1 (NS), 1‐844. Bootstrap values of 1000 were used.

## 3 RESULTS

### 3.1 Influenza virus isolation and subtype identification

In total, 8 H9N2 AIVs were isolated from 4534 samples collected from wild birds in Anhui province, China, from 2014 to 2015. Six strains were obtained in 2014 and two in 2015. Seven viruses were isolated from Anseriformes; the remaining virus was from a pigeon (Table 1). Among the 8 strains, one virus which named (AH/L139) was an internal gene supporter for the H10N8 subtypes, as previously reported.^11^ In this study, we analyzed the other seven viruses in this study. The complete genome sequences of the seven H9N2 viruses generated in our study were submitted to the GenBank database(MG781063‐MG781118).

### 3.2 Phylogenetic analysis of genes encoding surface proteins

**Figure 1:**
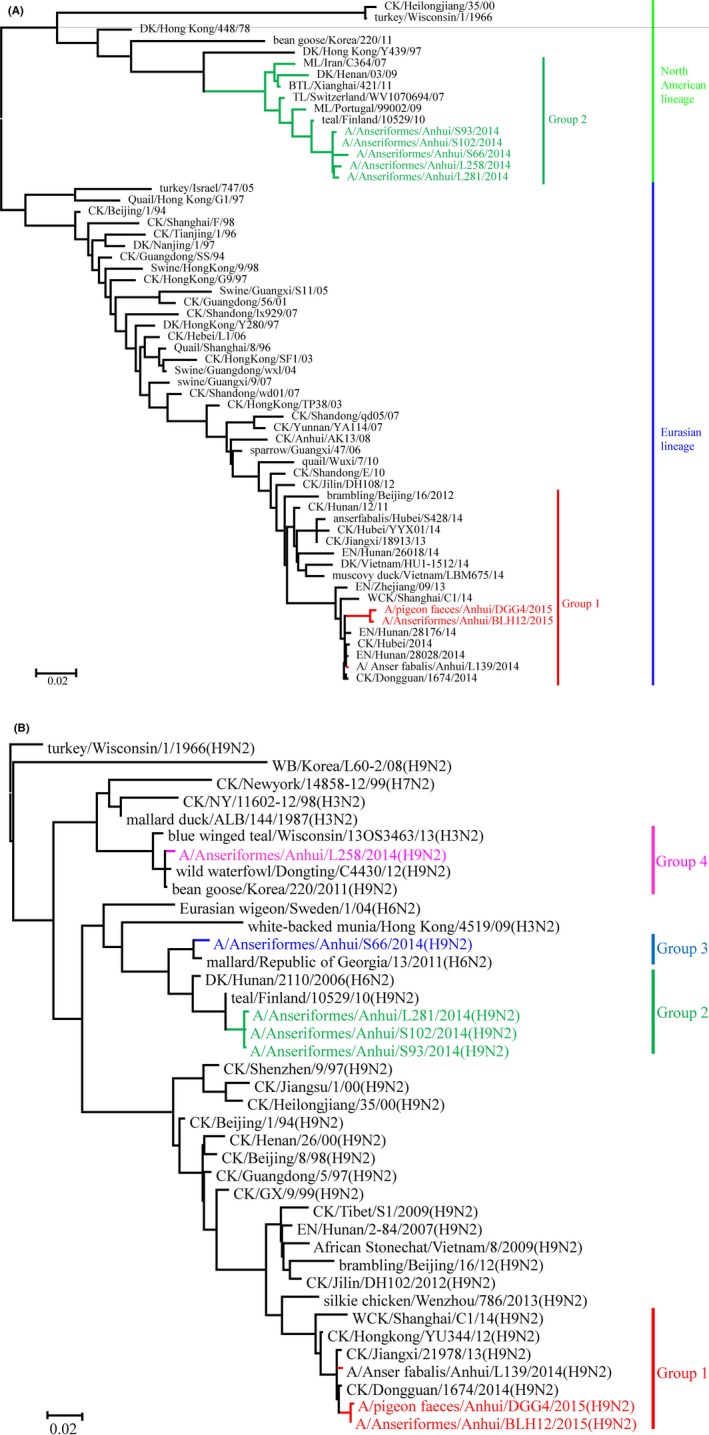
Phylogenetic analysis by maximum likelihood of the surface genes of H9 subtype AIVs isolated in 2014‐2015. The phylogenetic trees were generated using the mage 6.0 software package. The evolutionary history was inferred using the maximum likelihood method based on the Tamura‐Nei model. The tree with the highest log‐likelihood (−10 541.1792) is shown. The percentage of trees in which the associated taxa clustered together is shown next to the branches. Initial tree(s) for the heuristic search were obtained by applying the neighbor‐joining method to a matrix of pairwise distances estimated using the maximum composite likelihood (MCL) approach. The tree is drawn to scale, with branch lengths measured in the number of substitutions per site. The phylogenetic trees of the HA (A) and NA(B) genes used no root tree. The virus sequences listed in black were downloaded from available databases; viruses listed in red and green, blue, and pink were sequenced in this study. Abbreviations: CK, chicken; DK, duck; EN, environment; GS, goose; ML, mallard; SW, swine; WCK, wild chicken; WDK, wild duck

Phylogenetic analysis revealed that the HA genes of the seven H9 isolates could be separated into two geographically distinct lineages, Eurasian (group 1) and North American (group 2), based on a nucleic acid identity above 95% within each group (Figure 1A). Group 1 belonged to the Eurasian lineage, and group 2 belonged to the North American lineage. The homology within groups was 80.6%‐99.7% at the nucleic acid level. The two viruses in group 1 exhibited the highest sequence identity with (A/EN/Hunan/28176/14(H9N2)) in a GenBank BLAST alignment (https://blast.ncbi.nlm.nih.gov/Blast.cgi?PROGRAM=blastnPAGE_TYPE=BlastSearchLINK_LOC=blasthome). The other five viruses, which belonged to the North American lineage, had the highest sequence identity with (A/teal/Finland/10529/10(H9N2)) (Figure 1A).

The 7 N2 *NA* genes exhibited greater diversity than the *HA* genes and clustered into 4 groups based on nucleic identity above 95%. Viruses in groups 1, 2, and 3 belonged to the Eurasian lineage. There were three viruses in group 1, and all had the highest identity with (A/teal/Finland/10529/10(H9N2)); there was one virus (AH/S66) in group 2, and it had the highest identity with (A/mallard/Republic of Georgia/13/2011(H6N2)); and there were two viruses in group 3, both of which had the highest identity with (A/CK/Dongguan/1674/2014(H9N2)). AH/L258 in group 4, which belonged to the North American lineage, had the highest identity with (A/bean goose/Korea/220/2011(H9N2)). The homology of the nucleotides and amino acids was 83.0%‐99.8% and 84.%‐99.6%, respectively (Figure 1B).

### 3.3 Phylogenetic analysis of the internal genes

To better understand the evolution of the H9 AIVs isolated from wild birds in Anhui province, all six internal genes of the 7 novel genotypes identifies in this survey were phylogenetically analyzed as whole. Indications of the reassortment of two geographical lineages were also present in the internal genes. All of the internal genes exhibited a Eurasian lineage, except the NS gene of AH/S93, AH/S102, AH/L258, and AH/L281, which belonged to the North American lineage. The internal genes exhibited diversity. Eight segments of seven viruses comprised six genotypes based on their identity values (>95%) (Table 2).

**Table 2:**
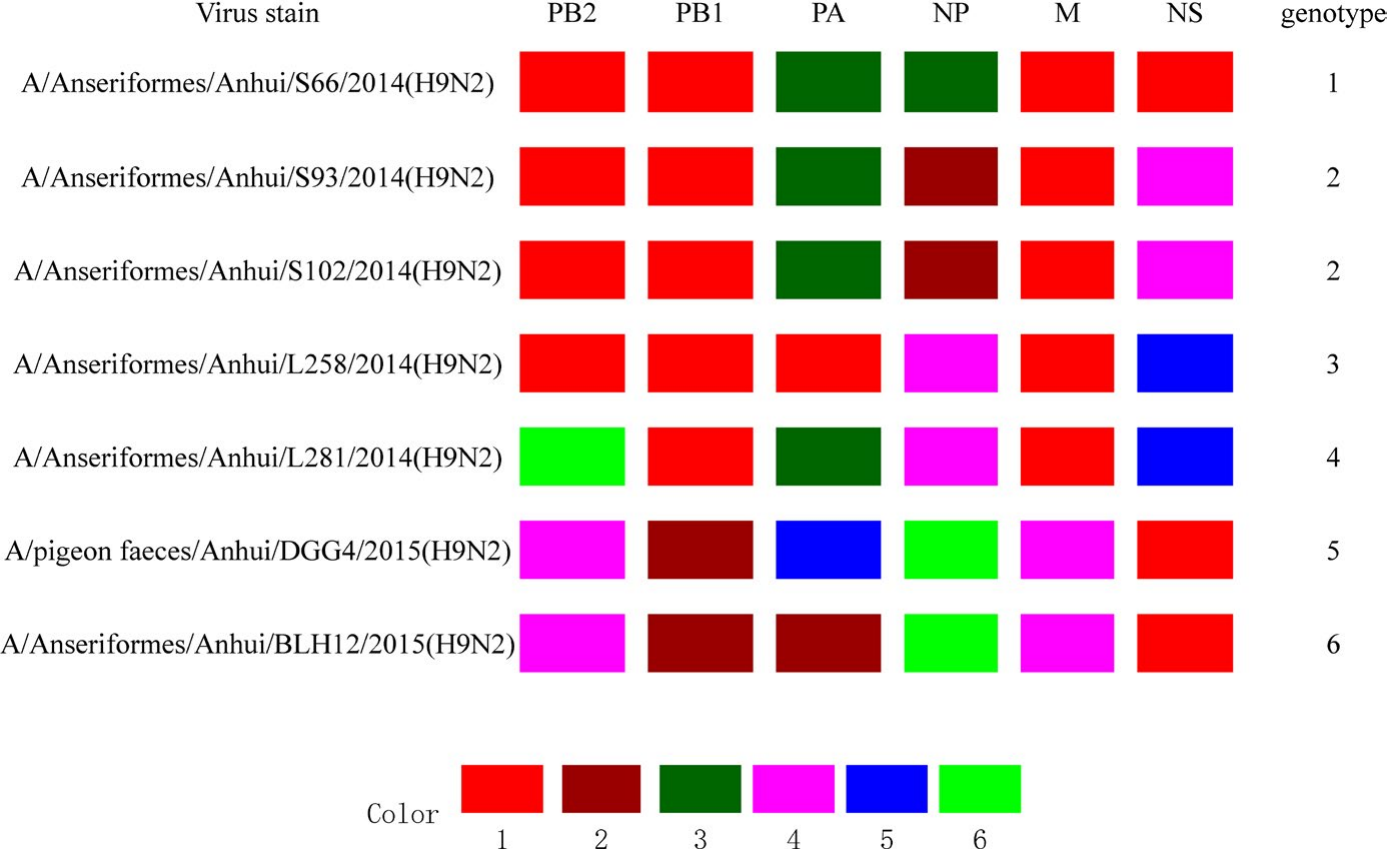
The genotypes of 7 H9N2 AIVs

**Figure 2:**
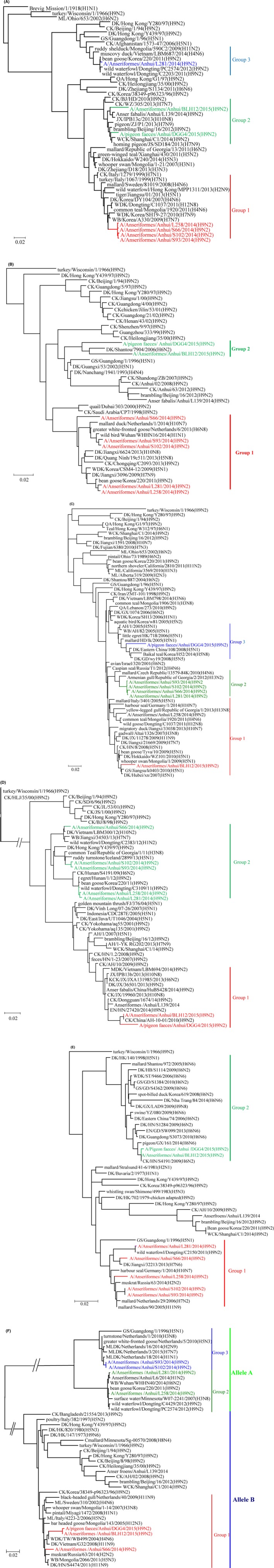
Phylogenetic analysis by maximum likelihood of the internal genes of H1 subtype AIVs isolated in 2014‐2015. (A) PB2, (B) PB1, (C) PA, (D) NP, (E) M, and (F) NS. The colors of the virus names in the PB2, PB1, PA, NP, M, and NS trees match those used in the genotype table. The phylogenetic trees of internal genes were rooted to A/Spanish/1/1918 (H1N1), with the exception of NS genes. The sequences of viruses listed in black were downloaded from GenBank; viruses listed in red, blue, and green were sequenced in this study. Abbreviations: CK, chicken; DK, duck; EN, environment; GS, goose; ML, mallard; SW, swine; WDK, wild duck

Based on nucleic acid identity, the PB2 and NS genes were divided into three groups. The PB1 and M genes were classified into two groups. The PA and NP genes were classified into four groups. The respective nucleotide and amino acid homologies from the 7 H9 isolates in this study were 90.7%‐99.3% and 98.0%‐100% for the PB2 genes, 89.7%‐99.4% and 93.7%‐99.6% for the PB1 genes, 92.7%‐99.8% and 96.4%‐99.7% for the PA genes, 90.7%‐99.5% and 98.0%‐100% for the NP genes, 92.2%‐100% and 90.2%‐100% for the M genes, and 70.6%‐99.3% and 62.3%‐98.6% for the NS genes (Figure 2A‐F).

**Figure 3:**
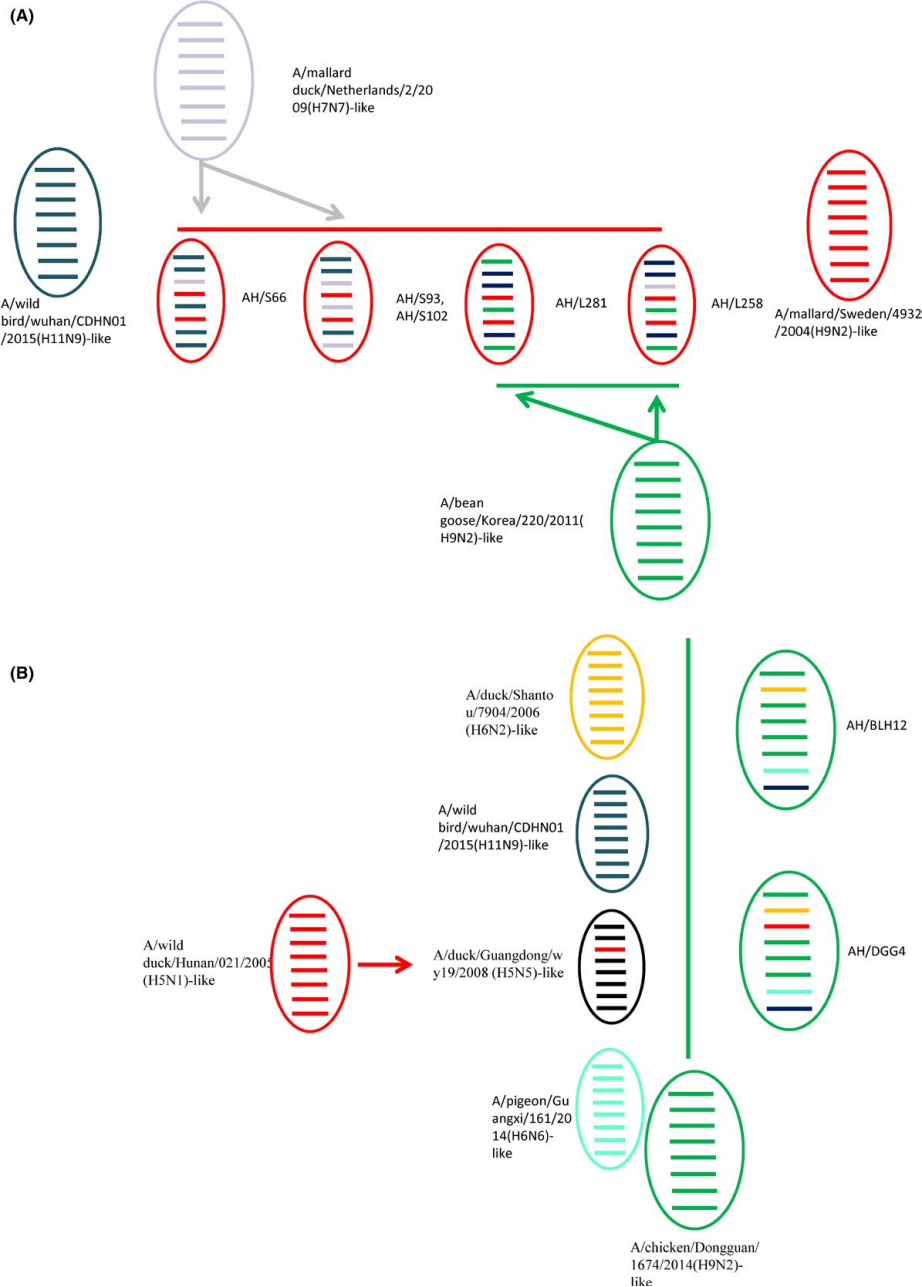
Genotypes of the H9N2 AIVs**.** The eight gene segments (from top to bottom) in each virus are PB2, PB1, PA, HA, NP, NA, M, and NS. Each color represents a separate source background. This illustration is based on the nucleotide distance comparison and phylogenetic analysis

The viruses isolated from wild birds’ samples showed genetic diversity. These seven viruses formed six genotypes. Virus AH/S66 designated genotype 1 was a reassortant virus containing PB2, PB1, NP, M, and NS genes from an A/wild bird/Wuhan/CDHN01/2015(H11N9)‐like virus gene pool and a PA gene from an A/mallard duck/Netherlands/2/2009(H7N7)‐like virus gene pool (Figure 3A). AH/S93 and AH/S102 designated genotype 2 were reassortants containing PB2, PB1, PA, and M genes from an A/wild bird/Wuhan/CDHN01/2015(H11N9)‐like virus gene pool and NP and NS from an A/mallard duck/Netherlands/2/2009(H7N7)‐like virus gene pool. AH/L258 of genotype 3 was a triple‐reassortant, containing PB2, PB1, and M genes from an A/wild bird/Wuhan/CDHN01/2015(H11N9)‐like virus gene pool; a PA gene from an A/mallard duck/Netherlands/2/2009(H7N7)‐like virus gene pool; and NP and NS genes from an A/bean goose/Korea/220/2011(H9N2)‐like virus gene pool. AH/L281 of genotype 4 was a reassortant, containing PB1, PA, and M genes from an A/wild bird/Wuhan/CDHN01/2015(H11N9)‐like virus gene pool and PB2, NP, and NS genes from an A/bean goose/Korea/220/2011(H9N2)‐like virus gene pool (Figure 3A). AH/BLH12 and AH/DGG4 were designated genotypes 5 and 6, respectively, and had complex gene origins but differed only in the PA gene. Their PB2, NP, and PA genes were from A/chicken/Dongguan/1674/2014(H9N2). Their NS genes were from an A/wild bird/Wuhan/CDHN01/2015(H11N9)‐like virus. Their PB1 gene had high identity (94%) with A/duck/Shantou/7904/2006(H6N2). The PA gene of AH/DGG4 may have originated from an A/wild duck/Hunan/021/2005 (H5N1)‐like gene pool (Figure 3B). Thus, all H9N2 viruses were showed different internal gene segments.

### 3.4 Molecular characterization of viral genes

Adaptive mutations in viral proteins are important for AIVs to cross species barriers and infect mammalian hosts.^12^ To determine whether the H9N2 viruses in this study had acquired genetic markers associated with mammalian pathogenicity, virulence, and adaptation to new hosts, we analyzed the whole‐genome sequences of all seven viruses. Overall, these viruses all had many potential N‐X‐T/S glycosylation sites (X is an amino acid other than P). The sites differed between the Eurasian and North American lineages. The viruses in the Eurasian lineage had 305‐308 potential glycosylation in their HA cleavage sites (A/pigeon feces/Anhui/DGG4/2015 and A/Anseriformes/Anhui/BLH12/2015); the viruses in the North American lineage had 210‐212 potential glycosylation sites and had deleted 305‐308 potential glycosylation sites (A/Anseriformes/Anhui/S66/2014, A/Anseriformes/Anhui/S93/2014, A/Anseriformes/Anhui/S102/2014, A/Anseriformes/Anhui/L258/2014, and A/Anseriformes/Anhui/L281/2014). None of the strains had consecutive basic amino acids in their motifs, and all conformed to the criteria for low pathogenicity AIVs.

The amino acid substitutions Q226L and G228S (H3 numbering, which is used throughout the manuscript) favor the affinity of influenza viruses for human‐type receptors.^13^, ^14^ Necklace deletion in the NA gene confers enhanced virus lethality in mice.^15^ The aforementioned characteristic changes were detected here: two viruses, AH/BLH12 and AH/DGG4, not only showed Q226L receptor binding changes but also had NA gene necklace deletions at amino acids 63‐65.

The influenza virus PB2 protein has several reported mutations that contribute to virulence and adaptation in mammalian hosts,^16^ including 89V,^17^ 271A, 627K, and 701N.^17^, ^18^, ^19^ Two viruses, AH/BLH12 and AH/DGG4, showed the E627K mutation in their PB2 proteins, and the other five strains had 271T, 627E, or 701D mutations. The substitution PB2‐A588V may represent a new strategy for AIV adaptation to mammalian hosts.^16^ The seven H9 strains in this study all had no substitutions in PB2‐A588V. The amino acid 292V/I is conserved in human and avian isolates^20^; therefore, 292V in the PB2 protein might be another important residue for the mammalian adaptation of AIVs.^16^ Here, two viruses, AH/BLH12 and AH/DGG4, had the 292V variant in the PB2 protein.

None of the seven H9 viruses had a Y436H substitution in the PB1 protein or T515A substitution in the PA protein, which suggested low pathogenicity to mammalian and avian hosts. An N66S substitution was found in the PB1‐F2 protein of all the H9 strains in this study, which is associated with the increased virulence of the 1918 pandemic virus and the highly pathogenic AI H5N1 virus in mice and ferrets.^21^, ^22^ The mutations N30D and T215A in the M1 protein and some had P42S in the NS1 protein suggest the viruses would exhibit increased virulence in mammals. No amino acid substitutions were found in the M2 transmembrane domain, suggesting that this virus strain is sensitive to M2 ion channel inhibitors.^23^ The S31N amino acid substitution in the M2 protein was not present, indicating that these viral strains were sensitive to amantadine inhibitors.^24^ The virulence of influenza viruses in humans is related to their resistance to the antiviral effects of cytokines, such as interferon (IFN), and the D92E mutation in the NS1 protein increases resistance to IFN.^25^ However, no mutations at residue 92 of NS1 were observed in this study.

## 4 DISCUSSIONS

H9N2 AIV is widely distributed in different regions of China and occasionally jumps hosts and reassorts with other subtypes of influenza virus, posing a severe public health threat.^26^ This AIV subtype has an impact that is significantly more severe than that observed in previous years not only because it is widespread but also because it is a donor of internal genes and undergoes extensive reassortment with different subtypes of avian AIVs, including HPAI H5N1 and H7N3.^27^ Recently, H9N2 has contributed internal genes to H7N9 and H10N8 viruses that cause severe human respiratory infections. During surveillance, we isolated one strain (AH/L139) whose six internal genes had high identity with H10N8 and H7N9, suggesting that the role of H9N2 AIVs as donors of internal genes cannot be ignored.^11^

The PB2‐K627 residue is a mammalian marker that is usually present in highly pathogenic AIVs (HPAIVs) and appears in mammalian‐adapted viruses.^28^ Importantly, a novel avian influenza A H7N9 virus infecting humans was identified in China.^29^, ^30^ This was the first time that the H7N9 subtype infected people and caused fatalities, with 402 confirmed cases and 146 deaths as of April 8, 2014.^31^, ^32^ Phylogenetic analyses have shown that the virus's six internal genes probably originated from H9N2 isolated from chickens^5^, ^6^. In H9N2 isolated from humans, a key signature amino acid at position 627 in PB2 was mutated to lysine,^5^, ^6^ which is associated with mammalian adaptation and increased virulence of the highly pathogenic avian influenza A (H5N1, H7N7, and H9N2) virus.^5^, ^6^ In December of the same year, Chinese health officials confirmed the first human case of avian influenza A H10N8 virus infection. The H10N8 virus contains the well‐characterized mammalian adaptation PB2 627K, and all internal gene segments (PB2, PB1, PA, NP, M, and NS) are derived from H9N2 viruses. Thus, this gene cassette might be a genetic platform for new strains with zoonotic potential.^33^ More evidence has demonstrated that PB2 residue 627 is a key determinant of host range and virulence for influenza A viruses,^34^ and the PB2 E627K mutation directly increases the enzyme activity of the polymerase that facilitates virus growth in vitro.^35^ In the H5N1 virus, PB2 E627K mutation is associated with systemic infection and impaired T‐cell activation in mice.^36^ Adaptation of H9N2 to mice is associated with multiple amino acid substitutions, including PB2 E627K.^37^

Here, we were interested in the two H9N2 virus strains (AH/DGG4 and AH/BLH12) isolated from fecal samples of pigeon and Anseriformes in Anhui in 2015, which had E627K mutations in their PB2 protein. This is the first report of a high pathogenicity mutation in wild birds, which suggests that influenza virus in wild birds, especially the H9N2 subtype, is undergoing a change from low pathogenicity to high pathogenicity. The HA protein of both strains demonstrates the ability to bind to human receptors (226L) as well as the deletion of certain amino acids in the neck of the NA protein, thus enhancing the pathogenicity of these strains in mice. Furthermore, we compared the corresponding amino acid sequences of internal genes and found amino acid changes associated with pathogenicity, IFN antagonism, and transmissibility in the amino acid sequences of PB2, PB1, M1, NS1, and PA.

H9N2 AIVs are widely distributed across the world; however, they primarily exist in poultry in Asia and are some of the most prevalent AIVs in poultry in mainland China. The persistent prevalence of H9N2 AIVs in poultry not only causes serious harm to the poultry industry but also constitutes an increasingly serious threat to human health. The extensive and stable presence of this subtype of AIV in wild birds provides favorable conditions for the emergence of a novel virus with pathogenicity to humans in China. These viruses are continually evolving due to viral mutation, recombination, and reassortment. The potential role of wild birds in the circumpolar circulation of influenza viruses points to the need to increase our knowledge of connectedness between migratory waterfowl populations originating from different wintering areas in the vast circumpolar (sub)arctic breeding areas. Surveillance of waterfowl at the crossroads of migratory flyways to wintering areas in Europe, Asia, and North America will inform epidemiological risk analysis and provide early warning of specific HPAI threats to poultry, and potentially human, health. Continued surveillance is required in wild birds, especially migrant birds in eastern China.

Wild birds serve as a huge reservoir and effective communicator of influenza viruses, playing a significant role in promoting virus spread and recombination, in particular during the seasonal migration of migratory birds and their close contact with local sentinel chickens and/or ducks. Wild birds containing an internal gene complex, enabling the generation of novel pathogenic viruses via gene rearrangement with other influenza viruses, will be threaten to human health in the future. Therefore, strengthening the AIV surveillance of wild birds will enable us to understand the presence and prevalence of influenza viruses and provide scientific evidence for the prevention and control of AIVs.

## CONFLICT OF INTEREST

None of the authors have a conflict of interest.
